# Femoral and Lateral Femoral Cutaneous Nerve Block as Anesthesia for High-Risk Intertrochanteric Fracture Repair Patients

**DOI:** 10.3390/jcm11133708

**Published:** 2022-06-27

**Authors:** Jakub Klimkiewicz, Anna Klimkiewicz, Mateusz Gutowski, Bartosz Rustecki, Dymitr Kochanowski, Robert Ryczek, Arkadiusz Lubas

**Affiliations:** 1 Department of Anesthesiology and Intensive Care, Military Institute of Medicine, 04-141 Warsaw, Poland; jklimkiewicz@wim.mil.pl (J.K.); mgutowski@wim.mil.pl (M.G.); brustecki@wim.mil.pl (B.R.); dcochanovschi@wim.mil.pl (D.K.); 2 Department of Psychiatry, Medical University of Warsaw, 00-665 Warsaw, Poland; anna.klimkiewicz@wum.edu.pl; 3 Department of Cardiology, Military Institute of Medicine, 04-141 Warsaw, Poland; rryczek@wim.mil.pl; 4 Department of Internal Diseases Nephrology and Dialysis, Military Institute of Medicine, 04-141 Warsaw, Poland

**Keywords:** femoral nerve block, intertrochanteric fracture, lateral femoral cutaneous nerve block

## Abstract

Introduction. Intertrochanteric fracture (IF) is a common injury among the elderly. Due to significant comorbidities, anesthesia for IF repair may be challenging. The authors propose femoral nerve block together with a lateral femoral cutaneous nerve block and sedation as an anesthetic technique for most severe cases of IF with contraindications to spinal anesthesia. Methods. In total, 61 patients were enrolled prospectively in a study, 19 received general anesthesia (GA group), 22 spinal anesthesia (SA group), and 20 nerve blocks with sedation (PNB group). Results. Groups were comparable in terms of age, gender, independence, and several comorbidities: diabetes, obesity, underweight, cardiovascular, and cerebrovascular incidents in the past, dementia, chronic obstructive pulmonary disease, and frailty. Heart failure (*p* = 0.033), hemoglobin < 10 g/dL (*p* = 0.001) and eGFR < 30 mL/min (*p* = 0.039) were more frequent in PNB group. PNB group had higher American Society of Anesthesiologists (ASA) (*p* < 0.001), Nottingham Hip Fracture Score (NHFS) (*p* < 0.001), and Charlson Comorbidity Index (CCI) (*p* = 0.002) scales scores, and lower probability of 10-year survival according to CCI (*p* = 0.012). GA group had more frequent active malignancy (*p* = 0.041). GA and PNB groups had a higher frequency of hemostasis disorder (*p* < 0.001). Surgery was completed under the scheduled anesthesia technique. Survival, frequency of cardio and cerebrovascular incidents after surgery, loss of independence, and postoperative delirium were comparable between groups, as well as the length of postoperative stay. Conclusions. Surgical repair of intertrochanteric fracture with intramedullary nailing system among elderly, frail, and sick patients can be conducted under peripheral nerve block. FNB and LFCNB in the combination is a viable option for IT fracture repair.

## 1. Introduction

Intertrochanteric fracture (IF) is one of the most common injuries requiring surgical treatment. Anesthesia for repairing that kind of fracture is an especially challenging problem for anesthesiologists due to the advanced age of patients and their significant comorbidities [1]. Regional anesthesia, including femoral nerve block (FNB) and lateral femoral cutaneous nerve block (LFCNB) is widely used for pain management among patients with hip fractures [2,3]. Among patients with hip fractures, in-hospital mortality varies from 1.6% to 9.7% [4,5], 30-day mortality varies from 6.6% to 10.9% [6,7] and 1-year mortality is reported to range from 26% to 30.8% [8,9]. Morbidity is also high and varies from 17.0% to 49.6% [5,10]. Discharged patients often suffer from impaired mobility and loss of independence and require 24-h care. This causes IF to be an important public health problem [11].

The best timing for the surgery is known to be the day of admission or the day after admission [12,13]. Surgery may be delayed because of patient or administration-related factors. Only major health problems such as anticoagulation, volume depletion, electrolyte imbalance, uncontrolled diabetes, uncontrolled heart failure, correctable cardiac arrhythmia or ischemia, acute chest infection, and exacerbation of chronic chest conditions can be accepted causes of delay [12,13,14,15]. There is strong evidence that shortening the time from admission to surgery decreases the rate of complications and mortality [16,17].

When surgery is to be performed in the postulated time frame, an anesthesiologist often faces the patient with poorly controlled severe systemic diseases and new medical problems due to the fracture.

Spinal anesthesia (SA) can be considered for IF repair. However, it is not always possible due to coagulopathies, low platelet count, antiplatelet drug administration, systemic anticoagulation, problems with proper patient positioning, patient refusal, and the inability of the patient to cooperate. Current guidelines recommend postponing invasive spine procedures and neuraxial blocks when anticoagulants and antiplatelet medications are used [18,19,20]. The essential time for cessation of activity in most anticoagulants and antiplatelet drugs significantly exceeds the window of time that is proposed for trauma hip surgery.

When SA is contraindicated or not applicable, general anesthesia (GA) may be administered. Unfortunately, this raises concerns about complications associated with hypotension induced with anesthetics such as stroke, acute coronary syndrome, and postoperative cognitive dysfunction (POCD) [21,22]. Moreover, weaning from mechanical ventilation may be problematic because of atelectasis, chest infection, muscle weakness, and prolonged clearance from anesthetics.

Bearing in mind the timing of surgery and the patient’s comorbidities, the question arises whether other anesthesia techniques are helpful for IF repair? In our opinion, an alternative solution would be FNB with LFCNB and sedation. Those can be done regardless of patient hemostasis. It hardly affects the circulatory and respiratory systems. We use that anesthesia technique in our center in patients scheduled for IF repair with inadequate control of systemic diseases, poor medical conditions, and contraindications to neuraxial block.

We perform this technique to sufficiently complete the procedure. The possibility of surgery at all, or especially in the recommended window of time after admission, compensates for the shortcoming of the anesthesia protocol advocated in this paper.

As we have used these PNB for IT hip fracture repair in the past, we set out to investigate whether the combination of FNB and LFCNB with sedation was a viable anesthetic alternative to GA or SA in high-risk patients undergoing IT fracture repair.

## 2. Material and Methods

Approval from the Bioethics Committee at the Medical Institute of Medicine (136/WIM/2018) was obtained. In addition, this research program was registered in Clinical Trials (NCT04470115).

### 2.1. Patients

A total of 61 patients were prospectively included in this observational study between June 2019 and January 2020. The inclusion criterion was an intertrochanteric fracture of the femur scheduled for surgical repair with a gamma3 intramedullary nailing system by Stryker. All patients were operated on by one orthopedic team in the care center of highest reference (Military Institute of Medicine, Warsaw, Poland). Anesthesia was provided by 3 attending physicians (JK, BR, and DK). A postoperative survey, including the occurrence of postoperative delirium, was done by an anesthesia trainee (MG). Previously, the authors had attempted to perform this in the randomized clinical trial protocol, but full randomization appeared not to be in the best interest of patients. Anesthesia technique was at the discretion of the anesthesia provider.

### 2.2. Anesthesia Technique

#### 2.2.1. Peripheral Nerve Blocks

We use a Sonosite Edge ultrasound machine, linear high-frequency transducer, 22G block needle, and ropivacaine 0.75%. In extremely frail patients, we reduce the volume and/or concentration to 0.5% not to exceed the recommended dose of local anesthetic.

Procedure: The patient is placed in a supine position. When a peripheral iv line is inserted and monitoring (SpO2, NIBP, ECG) is commenced, 50 mcg to 100 mcg of fentanyl iv is administered. Then the nerve block is performed. The groin is prepped and draped in a sterile fashion. The femoral nerve (FN) is identified laterally to the femoral artery below the fascia iliaca. The needle is inserted in-plane, from lateral to medial. Once the needle tip is adjacent to the nerve, and after careful aspiration, 1–2 mL of local anesthetic is injected to confirm the proper needle placement. Then we administer 15 to 17 mL of local anesthetic depending on the patient’s body mass, nerve visibility, or presence of a hematoma. After good anesthetic spread surrounding FN, we identify lateral femoral cutaneous nerve (LFCN) in its expected location 2–3 cm below the inguinal ligament, laterally to sartorius muscle, and usually inject 4 to 5 mL of local anesthetic. Approximately 20 min is enough to achieve anesthesia. When the patient reports that the hip is less painful while moving the limb, we gently palpate the hip. The patient is placed on the operating table when he reports no pain on palpation. Because of the patients’ frequent cognitive impairment, we usually do not perform pinprick tests. Oxygen 2–3 L/min is given via face mask to maintain Sp02, and the block is followed by sedation. Sedation is achieved with boluses of propofol to enable the procedure and maintain the patient in comfort. We aim to keep the depth of sedation at Ramsay’s sedation scale of 4. When it is possible, we use a loud verbal stimulus. If it is not, we use a gentle glabella tap to assess the depth of sedation. The patient is breathing spontaneously during the whole surgery. The capnography duct is placed under the face mask to monitor respiratory function. After the surgical field is prepared, the surgeon infiltrates the expected entry points of the intramedullary nail system with 0.5% lidocaine. Our orthopedic team usually fixes IF with an intramedullary nail (Gamma3 by Stryker). To enable the procedure, femur bone, skin, and subcutaneous tissue at 3 entry points, the greater trochanter, area of neck screw insertion, and area of distal fixation, need to be anesthetized. We administer 1 g of paracetamol during the surgery and a proton pump inhibitor. If the patient is unable to tolerate the procedure, we convert to GA, using sevoflurane in oxygen/air and some fentanyl as an analgesic. LMA or endotracheal tube is used to secure the airway.

#### 2.2.2. Spinal Anesthesia

Procedure: Patient is placed in lateral decubitus position with broken hip facing up. When a peripheral iv line is inserted, and monitoring (SpO2, NIBP, ECG) is commenced, we administer 50 mcg to 100 mcg of fentanyl iv. The lumbar area is prepped and draped in a sterile fashion. After gentle subcutaneous infiltration of the predicted point of needle insertion with 1% lidocaine, SA is achieved with the use of a 25G Quincke spinal needle and 10 to 15 mg of 0.5% isobaric bupivacaine. During the surgery, we administer 1 g of paracetamol and a proton pump inhibitor. There was no need to convert SA into GA among the studied group.

#### 2.2.3. General Anesthesia

Procedure: The patient is placed supine. When a peripheral iv line is inserted, and monitoring (SpO2, NIBP, ECG) is commenced, we perform induction of GA with fentanyl and propofol in a reduced dose. The airway is secured with LMA. Conduction of anesthesia is provided with sevoflurane in oxygen/air using age-adjusted MAC. During the surgery, we administer 1 g of paracetamol and a proton pump inhibitor. Ten patients from the GA group required radial artery cannulation and invasive blood pressure monitoring prior to induction due to reduced cardiovascular reserve and anticipated hemodynamic instability.

### 2.3. Clinical Tools

To assess perioperative risk, we used the Nottingham Hip Fracture Score (NHFS). NHFS was developed in 2008 by Maxwell and colleagues to predict 30-day mortality among patients with hip fractures [23]. Current literature suggests that NHFS is the best clinical tool to predict 30-day mortality among patients with hip fractures [24]. Designed in one surgical center, NHFS was validated in multicenter studies [25].

In the NHFS points are given for: age (≤65 years—0 points, 66–85 years—3 points, ≥86 years—4 points), male gender—1 point, low hemoglobin concentration at admission (≤10 g/dL)—1 point, living in an institution—1 point, more than two comorbidities (greater than, or equal to two)—1 point, presence of malignancy in the last 20 years—1 point, and low cognitive performance measured with Abbreviated Mental Test Score (AMTS of ≤6 out of 10)—1 point [23]. The AMTS was also used to detect cognitive dysfunction at admission. A cutoff ≤6 is used to indicate moderate-severe cognitive dysfunction associated with dementia [23,26]. NHFS score is positively correlated with 30-day mortality. Mortality was calculated with NHFS ranges from 0.7% for a patient receiving 0 points to 45% for a patient receiving a maximum of 10 points [24].

To diagnose the presence of frailty we used the Clinical Frailty Scale (CFS). CFS is based on appearance, ability to exercise, ability to manage daily routine and self-care, and need for assistance. CFS divides patients into 7 categories: very fit, well, well with treated comorbidity, vulnerable, mildly frail, moderately frail, and severely frail. Patients assigned to the last three groups were diagnosed with frailty for the purpose of our study [27].

By “hemostasis disorder” we mean occurrence of contraindication to spinal anesthesia due to antiplatelet agents, anticoagulation, low platelet count (<100,000/µL), prolonged INR or/and APTT.

By “independence” we mean living alone or with a spouse. By “dependency” we mean living in the institution (nursing home, long-term care facility) or living with children or grandchildren who provide care to a patient. By “loss of independence” we mean discharge of a previously self-caring patient to a long-term facility.

Postoperative delirium was diagnosed with the use of the Confusion Assessment Method for the Intensive Care Unit (CAM-ICU) [28]. CAM-ICU was found to be highly sensitive and specific in identifying delirium among patients with femur fracture [29].

To assess the presence of comorbidities, we used medical interviews and records in the information system. Based on diagnosed comorbidities, we calculated Charlson Comorbidity Index (CCI) [30]. We report two values connected with CCI-CCI: raw score and calculated probability of a 10-year survival. CCI was found to be a good predictor of the clinical outcome among patients undergoing hip fracture surgery [31,32,33].

### 2.4. Statistics

The results were presented as mean with standard deviation or the median with extreme values depending on the fulfillment of the criterion of normal distribution, which was checked with the Shapiro–Wilk test. If variables distribution differs between groups in normality, both the mean with standard deviation and the median with extreme values were presented. Nominal variables were presented as numbers. A comparison of the three nondependent groups was performed with the use of the Chi^2^ test if the variables were categorical, otherwise with the Kruskal–Wallis test due to the missing of the normal distribution. Two-tailed *p* < 0.05 was considered significant. To calculate the sample size, we qualified 8 consecutive patients to the GA group and the same quantity to the PNB group and then compared ASA scores between groups. To achieve a significant difference in ASA between groups (*p* < 0.05), a minimal sample size was estimated at 20 participants in each group for the power of the test = 0.9, and 15 for the power of the test = 0.8. For statistical analysis, the Statistica 12 software (StatSoft Inc., Cracow, Poland) was used.

## 3. Results

Demography and description of the studied group are presented in Table 1.

Patients (*n* = 61) were divided into three groups relating to the anesthesia technique—GA (general anesthesia, *n* = 19), SA (spinal anesthesia, *n* = 22), and PNB (peripheral nerve blocks, *n* = 20). Anesthesia technique was at the discretion of the anesthesia provider. We analyzed factors contributing to perioperative risk. Groups were comparable in terms of age, gender, frequency of living independently, diabetes, obesity, underweight, malignancy in the past 20 years, dementia, chronic obstructive pulmonary disease (COPD), and frailty. PNB group had higher ASA (*p* = 0.0001), NHFS (*p* = 0.0006), and CCI scores (0.002), a lower probability of 10-year survival according to CCI (*p* = 0.012). GA group had more frequent active malignancy than SA and PNB groups (*p* = 0.04). GA and PNB groups had a higher frequency of hemostasis disorder (*p* < 0.001). Data concerning perioperative risk are presented in Table 2 (continuous variable and categorical variables).

Results of the treatment are presented in Table 3. Surgery was completed under the scheduled anesthesia technique. Survival, frequency of cardio and cerebrovascular incidents after surgery, loss of independence, and postoperative delirium were comparable between groups, as well as the length of postoperative stay.

## 4. Discussion

The femoral nerve block, together with the lateral femoral cutaneous nerve block and sedation, can be an effective anesthetic technique for most severe cases of surgical treatment in intertrochanteric fracture with contraindications to SA. The presented procedure enables performing hip fracture surgery in patients with contraindications for GA or SA. It needs to be underlined that that type of surgery is considered a lifesaving intervention and an excellent method of pain relief. Our technique is feasible for patients who would have to wait for hemostasis normalization to receive SA. Additionally, we found this technique beneficial for patients with a poor reserve of the cardiovascular and respiratory systems. Before the introduction of the peripheral block technique, they would have likely received GA. Although we cannot name it contraindicated, we find GA not perfectly suitable for such patients. Keeping in mind that anesthetics may provoke hypotension, we prepared pumps with vasopressors, often placed in arterial and/or central lines. This makes induction of GA complicated and time-consuming in such cases, while the surgical procedure itself is rather short and minimally invasive. This makes turnover time longer and the operation room is less effectively used. The study was primarily planned as a fully randomized controlled trial. However, in the best interest of patients, those of them with more comorbidities and in worse clinical conditions eventually received more often peripheral nerve blocks. This explains the higher score in ASA, NHFS, and CCI observed in the PNB group due to more frequent dementia, heart failure, cardio and cerebrovascular events in the past, having eGFR ≤ 30 mL/min and hemoglobin level < 10 g/l. In addition, the PNB group received more frequently ≥4 medications to manage chronic diseases than GA and SA groups. This makes obtained results even more convincing: groups are comparable with each other, with the PNB group even more preoperatively burdened than the GA and SA groups, which was demonstrated with the use of ASA, NHFS, and CCI scales. The higher frequency of active malignancy in the GA group may be explained by the presence of metastatic tumors in the spine. Those patients were not offered SA. Additionally, a higher frequency of hemostasis disorders in groups GA and PNB can be explained by not offering SA to such patients. Anesthesia to IF fixation may be very challenging when it comes to ideal time, choice of technique, and procedure details. SA is a common option but may be contraindicated due to abnormal coagulation test results and ongoing antiplatelet or/and anticoagulation therapy. The authors find anticoagulation with Direct Oral Anti-Coagulant (DOAC) more challenging than anticoagulation with traditional vitamin-K antagonists because no routine laboratory tests exist to rule out the anticoagulation effect. Time-based criteria are usually used to rule out the anticoagulation effect, but it can take a long time in the group of elder, frail patients often with impaired renal function. The most common antiplatelet drug used among our patients is clopidogrel, which needs 5 to 7 days for its action to cease. That is why anticoagulation or antiplatelet therapy makes the use of SA not applicable when surgery is to be performed in a recommended time window [19,20]. Furthermore, positioning the patient with a hip fracture to proceed with spinal anesthesia can induce significant pain, regardless of if we put the patient in a sitting or lateral decubitus position. When SA is contraindicated or not applicable, GA can be administered. However, the use of GA raises concerns about possible complications, especially among elderlies. As mentioned before, patients with IF are frail and suffering from significant comorbidities, mainly: cardiovascular, respiratory, and renal diseases as well as dementia. Those make them at high risk of anesthetic-induced hypotension. Hypotension may lead to several adverse sequelae, with some potentially catastrophic such as stroke or myocardial infarction. Additionally, weaning from the ventilator after the surgery can be difficult.

At the very beginning, we used FNB only for postsurgical analgesia to reduce opioid administration. Further, we started to use it to facilitate patient positioning before spinal block. Then we used combined FNB and LFCNB with GA. We observed no need for additional fentanyl supplementation during the surgery. A single dose of fentanyl administered to facilitate LMA insertion or endotracheal intubation was the only opioid used during the whole procedure. Almeida et al. reported the use of fascia iliaca block (FIB) together with deep sedation to perform anesthesia for IF intramedullary nailing among three ASA IV patients [34]. Almeida chose this technique because of the poor clinical status together with P2Y12 inhibitors administration. Almeida used FIB as the main component of anesthesia. Noxious stimuli from regions innervated by sciatic and inferior subcostal nerves were managed by systemic analgesia with fentanyl and sedation with propofol. It`s worth noting that patients were breathing spontaneously through the procedure without the need for airway instrumentation or mask ventilation. The need for sedation was limited, as the authors report that Bispectral Index was above 75 for all patients. Almeida and associates conclude that choosing SA for these patients would cause a substantial delay in surgery. Kunisawa chose a fascia iliaca block together with a high dose of dexmedetomidine to provide surgical anesthesia to two individuals with hip fractures and severe cardiac conditions [35]. Similar to Almeida, Kunisawa reports that almost all areas of predicted skin incision and the major portion of fractured bone were anesthetized with the block. Surgical stimuli coming from not anesthetized regions of operated limbs were managed with dexmedetomidine infusion. Both cardiac function and respiratory status were stable throughout the surgical procedure. Fascia iliaca block is a safe and effective technique when used to provide pre- and postoperative analgesia among patients with hip fractures [36]. The novel approach was to use FIB as an anesthetic technique. We decided to combine FNB and LFCNB with sedation, not FIB, as we found this technique more selective when performed under ultrasound guidance. Johnston and associates report the use of FNB and LFCNB as a primary anesthetic technique among patients with hip fractures undergoing surgery [37]. In contrast to our method, they used a target-controlled infusion of propofol mixed with alfentanil as sedatives. We found no need for continuous opioid supplementation during the procedure; thus, sedation was based on propofol. A hip fracture may need even fixation with a total hip implant [38]. Because hip arthroplasty is performed in our center from posterolateral or posterior access, we did not find our technique of anesthesia to be suitable for this procedure, as did Johnston and colleagues.

The first limitation of our protocol comes from the fact that in our center, patients with IF after admission may have placed ankle traction with K-wire. Ankle traction is used to prevent further bone dislocation. During IF repair with an intramedullary nail, ankle traction is used to reduce the fracture. This causes nociceptive stimulation both in the hip region because of bone fragments movement, as well as in the ankle because of K-wire presence. The ankle is not anesthetized during our technique, so we administer more analgesics and sedatives to achieve desired sedation depth when traction with wire is used. When IF patients have no wire ankle traction placed, we do not need to administer additional sedatives during fracture reduction as pain stimuli come only from the hip joint, which is sufficiently anesthetized with our technique. Our second concern is that proper IF reduction sometimes requires inner thigh rotation. This makes the entry point of nailing lay more posterior, outside the anesthetized region. Therefore, we ask the surgeon to infiltrate the skin in the location of predicted nail entry. The third concern we had was whether to perform a sciatic nerve block as well or not, as the sciatic nerve contributes to sensory innervation of the femur and hip joint. Posterior approaches seem impractical in the case of IF due to patient positioning. Anterior approach to the sciatic nerve together with FNB, especially executed as SPEDI (Single Penetration Dual Injection) block, seemed to be the option, but we met technical difficulties in doing this block properly [39]. This was due to a change of anatomy because of hematoma, edema, and compulsory thigh position caused by pain. The last concern is the need for sedation when using this technique. As mentioned before, a substantial proportion of hip fracture patients suffer from cognitive impairment. Without sedation, they cannot tolerate the procedure because of severe anxiety, the compulsory position on the operating table, back pain, or pain from the other hip. Additionally, the surgical field is not perfectly anesthetized yet sufficient to complete the procedure. Sedation prevents the patient from the perception of breakthrough pain. Negative consequences of anesthetic or sedative agents’ administration are well known [22], but the authors find sedation necessary in this case. As we usually administer low doses of propofol—100 to 200 mg per case, its action ceases fast. Because we administer oxygen and monitor capnography, we do not observe severe respiratory depression or the need for mask ventilation. We administer boluses of propofol following the course of operation, e.g., before vigorous hammering or nail introduction. We find this way of propofol administration more practical than continuous infusion.

## 5. Conclusions

FNB with LFCNB and sedation is an effective technique of anesthesia in intertrochanteric repair surgery with an intramedullary nail. PNB with sedation is an additional anesthesia technique that can be used, when SA or GA seems not applicable or unsuitable. Currently, in our center, we use the presented technique as a salvage protocol of anesthesia which is offered to problematic patients with poor health conditions.

We plan to study if PNB (FNB, LFCNB) or new techniques such as pericapsular nerve group block can be applied for surgical repair of IF with other surgical systems.

## Figures and Tables

**Table 1:** Descriptive statistics.

Variable	Mean ±SD or Median [Min; Max] (*n* = 61)
Age (years)	86 [65; 97]
Hemoglobin (g/L)	11.9 ±1.8
eGFR (ml/min)	90 [24; 90]
ASA	3 [2; 4]
NHFS	6 [1; 8]
CCI	6 [2; 14]
CCI%	2 [0; 90]
Hospital stay after surgery [days]	7 [2; 60]

**Table 2:** Factors contributing to perioperative risk.

	General Anesthesia Mean ±SD; Median [Min; Max] (*n* = 19)	Spinal Anesthesia Mean ±SD; Median [Min; Max] (*n* = 22)	Peripheral Nerve Blocks Mean ±SD; Median [Min; Max] (*n* = 20)	*p*
Age (years)	83 ±7.8; 83 [65; 97]	84.4 ±8.1; 86 [68; 93]	87.7 ±5.5; 86 [79; 97]	0.252
Hemoglobin (g/L)	12.3 ±1.4; 12.6 [9.1; 14.2]	12.4 ±1.7; 12.6 [9.1; 15.7]	11.0 ±2.1; 10.5 [8.2; 14.8]	0.055
eGFR (ml/min)	90 [35; 90]	90 [45; 90]	50 [24; 90]	0.086
ASA	3 [2; 4]	3 [2; 4]	4 [2; 4]	<0.001
NHFS	5.3 ±1.6; 5 [1; 8]	5.1 ±0.9; 5 [3; 7]	6.6 ±1.1; 7 [4; 8]	<0.001
CCI	5.8 ±3.0; 5 [2; 14]	6.0 ±1.9; 6 [2; 9]	7.4 ±0.9; 8 [6; 8]	0.002
CCI%	21 [0; 90]	2 [0; 90]	0 [0; 2]	0.012
	**General Anesthesia** **(*n* = 19)**	**Spinal Anesthesia** **(*n* = 22)**	**Peripheral Nerve Blocks** **(*n* = 20)**	***p***
Gender (Male/Female)	6/13	4/18	5 (25%)	0.609
Dependency *n*(%)	3 (15.8%)	2 (9.1%)	2 (10%)	0.773
Diabetes *n*(%)	3 (15.8%)	2 (9.1%)	2 (10%)	0.573
Obesity BMI > 30 yes *n*(%)	2 (10.6%)	1 (4.5%)	2 (10%)	0.735
Underweight BMI <18.5 yes *n*(%)	1 (5.3%)	0	0	0.325
Cardiovascular and cerebrovascular incidents in past *n*(%)	8 (42.1%)	11 (50%)	15 (75%)	0.093
Active malignancy *n*(%)	4 (21.1%)	0	1 (5%)	0.041
Malignancy in last 20 years *n*(%)	0	1 (4.5%)	1 (5%)	0.624
Dementia *n*(%)	6 (31.6%)	7 (31.8%)	10 (50%)	0.383
Heart failure *n*(%)	10 (52.6%)	14 (63.6%)	18 (90%)	0.033
COPD *n*(%)	3 (15.8%)	1 (4.5%)	2 (10%)	0.483
Frailty *n*(%)	9 (47.4%)	16 (72.7%)	11 (55%)	0.233
More than 2 comorbidities *n*(%)	16 (84.2%)	18 (81.8%)	20 (100%)	0.141
More than 4 medications *n*(%)	14 (73.7%)	19 (86.2%)	20 (100%)	0.052
Hemostasis disorders *n*(%)	18 (94.7%)	22 (100%)	10 (50%)	<0.001
Hemoglobin < 10 g/dL *n*(%)	3 (15.8%)	1 (4.5%)	10 (50%)	0.001
eGFR <30 mL/min *n*(%)	0	0	3 (15%)	0.039

**Table 3:** Results of the treatment.

	General Anesthesia (*n* = 19)	Spinal Anesthesia (*n* = 22)	Peripheral Nerve Blocks (*n* = 20)	*p*
Death *n*(%)	1 (5.3%)	2 (9.1%)	1 (5%)	0.835
Cardiovascular and cerebrovascular events after surgery *n*(%)	0	3 (13.6%)	2 (10%)	0.266
Loss of independence *n*(%)	2 (10.6%)	5 (26.3%)	5 (25%)	0.427
Delirium *n*(%)	10 (52.6%)	9 (41%)	10 (50%)	0.728
Hospital stay after surgery [days] Median [min; max]	7 [5; 9]	7.5 [4; 60]	8 [2; 50]	0.153

## Data Availability

Not applicable.
